# Comparison of chemical profiles and effectiveness between Erxian decoction and mixtures of decoctions of its individual herbs: a novel approach for identification of the standard chemicals

**DOI:** 10.1186/s13020-016-0123-8

**Published:** 2017-01-03

**Authors:** H. P. Cheung, S. W. Wang, T. B. Ng, Y. B. Zhang, L. X. Lao, Z. J. Zhang, Y. Tong, F. W. S. Chung, S. C. W. Sze

**Affiliations:** 1 School of Chinese Medicine, Li Ka Shing Faculty of Medicine, The University of Hong Kong, 10 Sassoon Road, Pokfulam, Hong Kong, SAR China; 2 School of Biomedical Science, Faculty of Medicine, The Chinese University of Hong Kong, Shatin, N.T., Hong Kong, SAR China

**Keywords:** Standard chemicals, Chinese medicine formula, Erxian decoction, Menopause, Novel approach

## Abstract

**Background:**

Identification of bioactive standard chemicals is a major challenge in the study of the Chinese medicinal formula. In particular, the chemical components may interact differently depending on the preparative methods, therefore affecting the amounts of bioactive components and their pharmacological properties in the medicinal formula. With the use of Erxian decoction (EXD) as a study model—a  well-known Chinese medicinal formula for treating menopausal symptoms, a novel and rapid approach in seeking standard chemicals has been established by differentially comparing the HPLC profiles and the menopause-related biochemical parameters of combined decoction of EXD (EXD-C) and mixtures of the decoctions of its individual herbs (EXD-S).

**Methods:**

The levels of six chemicals, which exerted actions on the HPO axis, have been measured in EXD-C and EXD-S by HPLC. Twelve-month-old female Sprague-Dawley rats were employed and treated with EXD-C and EXD-S. Their endocrine functions after treatment were evaluated by determining the ovarian mRNA levels of aromatase, a key enzyme for estradiol biosynthesis. The effect of the antioxidant regimen was determined by the hepatic superoxide dismutase-1 (SOD), catalase (CAT) and glutathione peroxidase (GPx-1) mRNA levels.

**Results:**

The amounts of mangiferine, ferulic acid, jatrorrhizine and palmatine in EXD-S were twofold higher than those in EXD-C. EXD-S was more effective in stimulating ovarian aromatase and the expression of the antioxidant enzymes compared with EXD-C.

**Conclusion:**

Mangiferine, ferulic acid, jatrorrhizine and palmatine are suitable for use as standard chemicals for quality evaluation of EXD according to our approach. EXD-S could be more effective than EXD-C.

## Background

The clinical use of Chinese medicine (CM) formulas for treating chronic diseases and aging-related disorders has been gaining momentum in recent years. The multiple pharmacological properties of CM formula are proved to be contributed by the multiple bioactive chemicals in a CM formula through multiple mechanisms [1]. Thus, seeking bioactive and standard chemical is crucial for quality control evaluation of CM formulas. While the contemporary analytic techniques allow us to isolate and detect multiples chemical components from CM formulas simultaneously, identification of bioactive and standard chemicals remains a tedious task.

Due to the complexity of the chemicals in a CM formula, the potential interaction and the subsequent changes in the pharmacological properties have drawn the attention of many researchers in the field [1]. Interactions leading to changes in the chemical components of TCM may arise during the processing of herbal materials or during the decoction procedures [2]. Conventionally, different herbal materials in a Chinese medicinal formula are decocted together to yield the medicinal extract, which can be regarded as a “combined decoction”. To the contrary, individual herbs can be decocted separately and mixed together to compose a medicinal formula. This is particularly common in the recent development of herbal formulations, extracts of individual herbal material can be concentrated in the form of granules, and the medicinal formula can be reconstituted by mixing the corresponding amounts of granules [3]. The resulting decoction is thus regarded as a mixture of decoctions of separated individual herbs (separated decoction)”. However, due to the difference in their decoction processes, there will be variations in the chemical components of the “combined decoction” and “separated decoction”. It is known that during the decoction process, the chemicals from different herbs may interact to affect the solubility, conversion of chemical structures, or may lead to generation of new chemicals or precipitation [3]. For example, results obtained from a previous study revealed that the component of 1,5-dicaffeoylquinic acid in the Tibetan herb (*Saussurea laniceps*) was transesterificated into 1,3-dicaffeoylquinic acid during boiling in water [4]. These interactions may affect the amounts of bioactive components in the medicinal formula, and thus the pharmacological properties. On the one hand, the discrepancy between the chemical components and the pharmacological properties between “combined decoction” and “separated decoction” has raised concerns about the variation in quality and therapeutic efficacy of decoctions prepared using different methods. On the other hand, such discrepancy may hint the bioactive components contributing to the pharmacological properties, thus opening up the possibility of a novel and rapid approach for identification of the chemical standards of CM formulas for quality control purpose.

In this study, Erxian decoction (EXD), an anti-menopausal Chinese medicine formula, was selected as a study model to demonstrate the feasibility of our approach for identification of the chemical standards of CM formulas. Erxian decoction (EXD) is a popular TCM formula that has been clinically used for relieving menopausal syndrome for more than 60 years [5]. Our previous EXD studies have used a LC-DAD-ESI-MS/MS method in characterising the key chemical constitutents of EXD absorbed or metabolised in vivo during the treatment of menopausal syndromes [6]. Knowing that the causes of menopausal syndromes involves the hypothalamus-pituitary-ovary (HPO) axis, target compounds of this study were identified and differentiated by their presence in the major organs of the HPO axis (i.e. brain and ovary) and serum. Six chemicals from EXD that may contribute to relief of menopausal syndrome were selected: mangiferine, ferulic acid, icariin, jatrorrhizine, palmatine and berberine [6]. Therefore, the levels of mangiferine, ferulic acid, icariin, jatrorrhizine, palmatine and berberine in both EXD-C and EXD-S were determined and compared by HPLC profiles. Besides chemical analysis, it has been found in human that during menopause, the expression of aromatase, a key enzyme for ovarian estradiol production, and the activities of some antioxidant enzymes, including superoxide dismutase-1 (SOD1), and glutathione peroxidase (GPx-1), underwent a decrease leading to a decline of ovarian estrogen production and serum estrogen level [7]. In addition, our previous study also demonstrated that EXD relieved menopausal syndrome via up-regulation of the mRNA levels of ovarian aromatase and hepatic antioxidant enzymes catalase (CAT) in twelve-month-old naturally aging SD-rats with lower serum estradiol levels compared with those of 3-month-old young SD-rat [5]. Therefore, we evaluated and compared the pharmacological properties of EXD-C and EXD-S for alleviating menopause by measurement of their mRNA levels of ovarian aromatase and hepatic antioxidant enzymes SOD-1, CAT, and GPx-1 after drug treatment. Results and approach obtained from this study could be applied in quality control studies of other existing CM formulas, which ensure the use of high-quality CM formulas clinically. The following shows our approach for selecting the standard markers for quality control of CM formulas: (i) the standard markers should be present at target sites/organs in vivo; (ii) the standard markers should be associated with pharmacological and clinical effects of CM formulas; (iii) the amounts of standard markers should have twofold differences for different decoction processing (EXD-S: separate decoction of EXD vs EXD-C: combined decoction of EXD).

## Methods

### Herbal materials and preparation of different decoctions of EXD

One kilogram of the six component herbs of EXD namely *Herba Epimedii, Rhizoma Curculiginis, Radix Morindae officinalis, Cortex Phellodendri, Radix Anemarrhenae,* and *Radix Angelicae sinensis* (composition ratio = 12:12:10:10:9:9) were decocted together with distilled water in 10:1 (v/w) ratio at 100 °C for 1 h to prepare “combined decoction of EXD” (EXD-C), the extraction was repeated twice. The procedures were the same as those described in our previous publication [5]. For preparation of “mixtures of EXD individual herbs decoction” (EXD-S), the component herbs in the amounts according to above composition ratio of EXD-C were decocted separately instead and reconstituted afterward. The extraction was also repeated twice. Both of the herbal extracts, EXD-C and EXD-S, were filtered and lyophilized in a freeze drier (Labconco, Freezone). The dried powdered extracts were stored at −80 °C before use. The herbal materials were collected from various sources and their identity was confirmed by Dr YanBo Zhang (one of the authors), School of Chinese medicine, the University of Hong Kong.

### Quality control and high performance liquid chromatography (HPLC)

To evaluate the consistency of the quality of EXD-S and EXD-C extracts, three batches of 0.5 g powder of extracts were extracted with 10 ml 75% methanol in a water bath at 60 °C for 15 min, followed by ultrasonication for 30 min. The extracts were centrifuged at 15,700×*g* and filtered with 0.45 μm Millex® syringe filter (Millipore). Six standard chemicals namely mangiferine, ferulic acid, icariin, jatrorrhizine, palmatine and berberine which are well-known compounds in EXD [5] were employed for quantitation. The HPLC profiles of the EXD-S and EXD-C were generated using Water 600S HPLC system (Waters) with a reverse-phase column (XBridge® C18, 5 μl, 250 mm × 4.6 mm i.d., Waters, USA). The mobile phase consisted of acetonitrile (solvent A) and 0.05% SDS in 0.1% acetic acid (solvent B). A programmed gradient was used for elution with 5–30% A in 0–30 min, 30% A in 30–35 min, 30–50% A in 35–40 min, 50–55% A in 40–65 min. The injection volume was 10 μl and flow rate was 1 ml/min. The ultraviolet (UV) absorbance from 200 to 400 nm was measured with a diode array detector (DAD). Chromatograms were generated at 345 nm to observed most number of peaks. The peak integration and quantitation were analyzed with the Waters Empower 2 software (Waters), the procedures were the same as those described in our previous publication [5].

### Animals

Twelve-month old female Sprague-Dawley (SD)-rats with low serum estradiol levels were employed as the animal model [5]. Animals were purchased at the age of 8 months from the Laboratory Animal Units, the University of Hong Kong and housed at an ambient temperature of 24 °C with a relative humidity of 50–65% and 12-h light–dark cycles till the required age. The rats were acclimated for four months and their serum estradiol levels were monitored before the experiment. The experiments had been approved by the Committee on the Use of Live Animals in Teaching and Research (CULATR) of the Li Ka Shing Faculty of Medicine, the University of Hong Kong.

### Drug administration and organ harvesting

Rats were arbitrarily divided into six groups with ten animals each. EXD-S and EXD-C extracts dissolved in 2 ml of water (0.76 and 1.52 g/kg) were administered via gavage tubing daily for 6 weeks. The control group received an equal volume of water instead of drug. At the end of the experiment, the rats were euthanized by an intraperitoneal injection of pentobarbital (200 mg/kg). The ovaries and livers were collected and stored at −80 °C until further experiment.

### RNA extraction and quantitative real-time PCR

The RNA extraction and quantitative real-time PCR were performed according to the previous methods published by our group [5]. In brief, total RNA was isolated from the ovary and liver using the TRIZOL® reagent according to instructions of the manufacturer (Invitrogen Life Technologies). The purity and concentration of RNA were determined by the absorbance at 260/280 nm and at 260 nm, respectively. The cDNA was transcribed from 1 μg of total RNA using random hexamers (Promega) and reverse transcriptase II (Invitrogen Life Technologies) following the manufacturer’s instructions. Quantitative real-time PCR was performed for the expression of aromatase (Cyp19), CAT, SOD-1, glutathione peroxidase 1 (GPx-1) genes and beta-actin (β-actin) as housekeeping control using the Platinum® quantitative PCR SuperMIX-UDG (Invitrogen Life Technologies) in a final reaction volume of 25 μl in 0.25 X SYBR green (Molecular Probes®, Invitrogen Life Technologies) according to the manufacturer’s protocol. The sequences of the PCR primers are described in our previous study [5]. The target genes were amplified with the following programme: pre-incubation at 94 °C for 15 min, followed by 40 cycles of incubation at 94 °C for 20 s, 57 °C for 20 s and 72 °C for 20 s. Following the amplification process, a melting curve analysis was performed by raising the temperature from 72 to 95 °C at a rate of 1 °C/5 s to ensure the specificity of PCR products. Quantitation of PCR product was performed by comparing with the standard curve (plot of number of threshold cycle (Ct) value against log of standard amount with a series of 20-fold dilution), and the results were expressed as Ct value. Quantity of the target genes was normalized with the housekeeping gene β-actin for relative quantitation. The experiments were repeated in triplicate for analysis.

### Statistical analysis

For the peaks in HPLC profiles of EXD-S and EXD-C, relative standard deviation (RSD) was calculated. For PCR experiments, data were expressed as mean ± SEM. Statistically analysis was performed using One-way ANOVA followed by Tukey’s Multiple Comparison Test. A p value <0.05 in a comparison was considered statistically significant. Statistical analysis was performed with GraphPad Prism 4® software (GraphPad Software).

## Results

### HPLC profiles of EXD-S and EXD-C

**Fig. 1:**
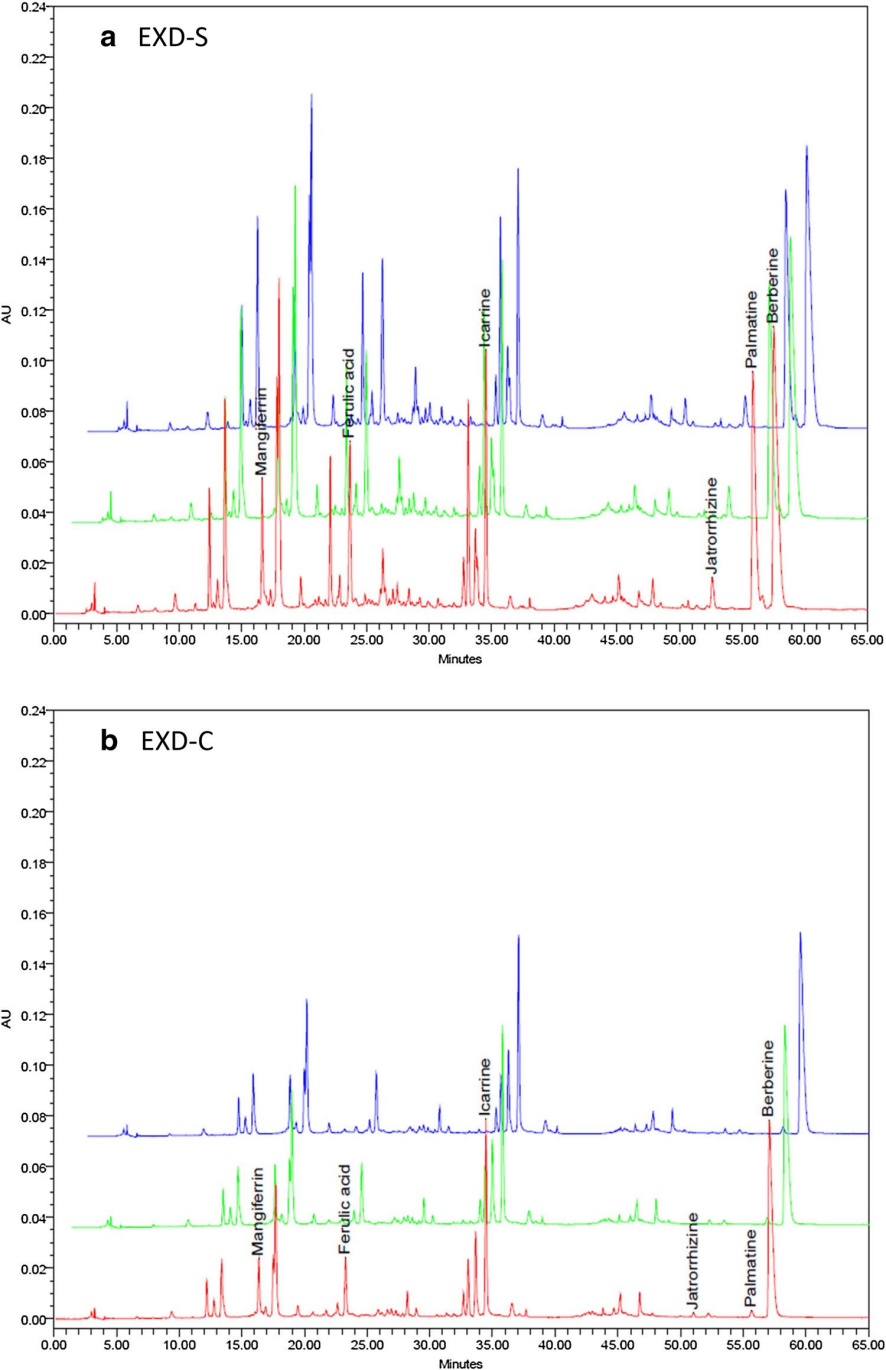
Overlaid HPLC chromatograms of **a** EXD-S [4] and **b** EXD-C from three repeated injections extracted at 345 nm. The peaks of six standard chemicals were annotated as mangiferine, ferulic acid, icariin, jatrorrhizine, palmatine and berberine, in a chorological order of retention time

**Fig. 2:**
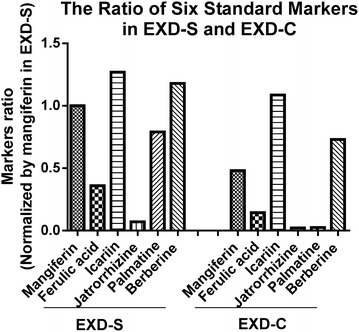
The ratio of six standard markers in EXD-S and EXD-C

**Fig. 3:**
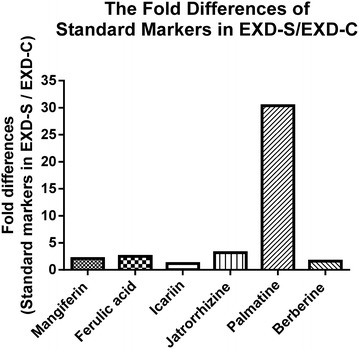
The fold differences of standard markers in EXD-S/EXD-C

The peaks from chromatograms generated at 345 nm show most detectable peaks were integrated. The chromatograms of EXD-S and EXD-C annotated with the six standard chemicals are shown in Fig. 1. Three batches of EXD-S and EXD-C were injected. The amounts of the six standard chemicals were determined from the standard curve and are listed in Table 1. The contents of all the six marker chemicals were found to decrease to different extents in EXD-C (Fig. 2). The content of mangiferin in EXD-S and EXD-C demonstrated a 2.09-fold difference. The decrease in content of three berberine-type alkaloids (jatrorrhizine, palmatine and berberine) in EXD-C varied from 3.44-old for jatrorrhizine, 30.17-fold for palmatine and 1.62-fold for berberine. The content of ferulic acid in EXD-C decreased by 2.46-fold and the amount of icariin showed a 1.17-fold decrease in EXD-C (Fig. 3). For all the six standard chemicals, the RSD values calculated were within 5%, indicating consistency in the quality of the sample injected and reproducibility of the HPLC profile, as well as excluding the influence of any unknown variability or instability found in the composition of the active constituents in the molecular investigation of the EXD extract.

**Table 1:** The contents of six standard chemicals of EXD in three injections of EXD-S and EXD-C

Injection	Mangiferin (mg/g)	Ferulic acid (mg/g)	Icariin (mg/g)	Jatrorrhizine (mg/g)	Palmatine (mg/g)	Berberine (mg/g)
EXD-S1	1.368	0.4871	1.731	0.1004	1.083	1.615
EXD-S2	1.371	0.4896	1.744	0.1010	1.090	1.617
EXD-S3	1.382	0.4996	1.745	0.1014	1.092	1.628
Mean	1.374	0.4921	1.740	0.1010	1.089	1.620
RSD (%)	0.57	1.34	0.46	0.48	0.46	0.43
EXD-C1	0.6581	0.1983	1.498	0.02865	0.03577	1.000
EXD-C2	0.6610	0.1980	1.493	0.03009	0.03661	1.001
EXD-C3	0.6583	0.2034	1.479	0.02947	0.03592	1.003
Mean	0.6591	0.1999	1.490	0.02940	0.03610	1.001
RSD (%)	0.24	1.53	0.65	2.45	1.23	0.17
Mean ratio	2.085	2.462	1.168	3.435	30.17	1.618

The results are expressed as mg or chemicals per g of EXD extract. RSD values were calculated for each chemical from three injections and the mean ratio represents the ratio of amount of chemicals in EXD-S to that of EXD-C

### Effects of EXD-S and EXD-C on expression of Cyp19, CAT, SOD and GPx-1 at transcriptional level

**Fig. 4:**
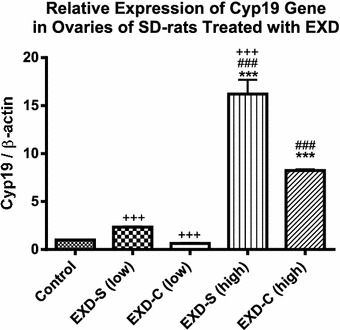
The relative expression of Cyp19 gene at transcriptional level in ovaries of SD-rats treated with different EXD decoctions. Data were normalized by control group and expressed as mean ± SEM. Control: control group (fed with water); EXD-S: SD-rats treated with separate decoction of EXD at 0.76 g/kg (low) and 1.52 g/kg (high); EXD-C: SD-rats treated with combined decoction of EXD at 0.76 g/kg (low) and 1.52 g/kg (high). ***p < 0.001 compared with Control;^###^p < 0.001 compared with EXD-C (low);^+++^p < 0.001 compared with EXD-C (high) (Tukey’s Multiple Comparison Test following One-way ANOVA) (n = 3)

After treatment with EXD-S and EXD-C for 6 weeks, the expression of ovarian Cyp19, SOD, CAT and hepatic GPx-1 was regulated differently. From the results, both treatments with EXD-S and EXD-C at high doses significantly stimulated the expression of ovarian Cyp19 gene, which encodes the key enzyme aromatase for estrogen secretion. (*p* < *0.01* compared with control group in Tukey’s Multiple Comparison Test following One-way ANOVA). The stimulatory effect on up-regulation of Cyp19 was most prominent in EXD-S at high dose, which is in line with our previous finding [5], in which the expression level of Cyp19 gene was significantly higher than that of EXD-C at high dose (*p* < *0.001* compared with control group in Tukey’s Multiple Comparison Test following One-way ANOVA) (Fig. 4).

**Fig. 5:**
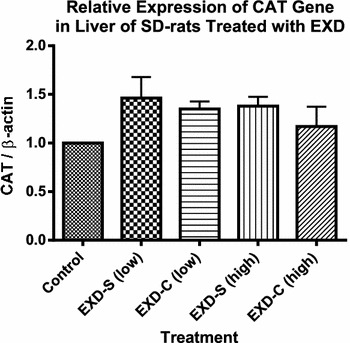
The relative expression of CAT gene at transcriptional level in livers of SD-rats treated with different EXD decoctions. Data were normalized by control group and expressed as mean ± SEM. Control: control group (fed with water); EXD-S: SD-rats treated with separate decoction of EXD at 0.76 g/kg (low) and 1.52 g/kg (high); EXD-C: SD-rats treated with combined decoction of EXD at 0.76 g/kg (low) and 1.52 g/kg (high). No statistical significances were detected among groups (Tukey’s Multiple Comparison Test following One-way ANOVA) (n = 3)

The effects of EXD-S and EXD-C were less prominent on the gene expression of hepatic antioxidant enzymes. The relative mRNA levels of CAT after treatment of EXD were slightly higher than that of control by around 1.5-fold, without statistical significance. EXD-S treatment at both dosages displayed a trend of increase in CAT expression compared with EXD-C, but again no significant differences were detected (Fig. 5).

**Fig. 6:**
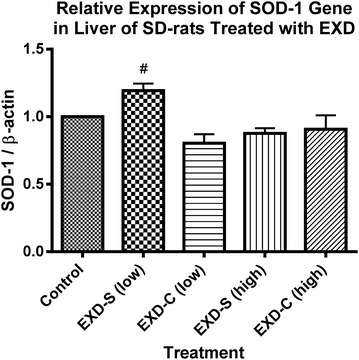
The relative expression of SOD-1 gene at transcriptional level in livers of SD-rats treated with different EXD decoctions. Data were normalized by control group and expressed as mean ± SEM. Control: control group (fed with water); EXD-S: SD-rats treated with separate decoction of EXD at 0.76 g/kg (low) and 1.52 g/kg (high); EXD-C: SD-rats treated with combined decoction of EXD at 0.76 g/kg (low) and 1.52 g/kg (high).^#^ *p* < *0.05* compared with EXD-C (low) (Tukey’s Multiple Comparison Test following One-way ANOVA) (n = 3)

**Fig. 7:**
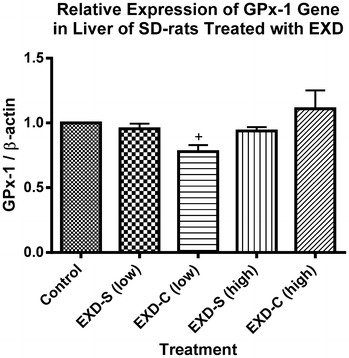
The relative expression of GPx-1 gene at transcriptional level in livers of SD-rats treated with different EXD decoctions. Data were normalized by control group and expressed as mean ± SEM. Control: control group (fed with water); EXD-S: SD-rats treated with separate decoction of EXD at 0.76 g/kg (low) and 1.52 g/kg (high); EXD-C: SD-rats treated with combined decoction of EXD at 0.76 g/kg (low) and 1.52 g/kg (high).^+^ *p* < *0.05* compared with EXD-C (high) (Tukey’s Multiple Comparison Test following One-way ANOVA) (n = 3)

The mRNA levels of SOD-1 and GPx-1 in all treatment groups were comparable to that of control. However, in EXD-S (low dose) treated group, the hepatic mRNA expression of SOD-1 was significantly higher than that of EXD-C (low dose) group (Fig. 6). EXD-S at high dose also displayed a tendency of increase in the mRNA level of hepatic GPx-1 compared with that of EXD-C groups, but such tendency was devoid of statistically significant difference (Fig. 7).

## Discussion

The effects of different decoction methods on chemical profiles of anti-menopausal EXD formula have been demonstrated by HPLC, with the six selected chemicals that have been present at HPO-axis in vivo revealed from our previous publication [6]. Results obtained from HPLC profile revealed that all the six chemicals including mangiferin, ferulic acid, icariin, jatrorrhizine, palmatine and berberine were higher in content in EXD-S than that in EXD-C (Figs. 2, 3), the HPLC profile of EXD-S is the same as that in our previous publication [5]. Such changes in chemical profiles may be due to the interaction of different components during the decoction process. For instance, the chemical components may enhanced the solubility of each other when decocted together thus increasing the final content of chemicals in the extract [8]. On the contrary, they may precipitate with each other forming insoluble complex leading to loss of bioactive components [8]. It is known that alkaloids like berberine, palmatine and jatrorrhizine would form precipitate with the flavone baicalin [9]. Alkaloids may also precipitate with organic acids forming insoluble salts [8]. It is possible that the alkaloids species in combined decoction of EXD (EXD-C) may precipitate with organic acid from its different ingredient herbs like ferulic acid, flavonoid compounds such as icariin or other undetected flavone species and are lost from EXD-C. Also, bioactive components can be converted by chemical reactions like hydrolysis of glycosides. In the combined decoction of EXD (EXD-C), hydrolysis may be facilitated to remove the sugar units from the flavonoids glycoside icariin, leading to decrease in its content [2].

Mangiferine, ferulic acid, jatrorrhizine and palmatine were confirmed as the key chemical markers for quality control of anti-menopausal EXD according to our proposed approach. Because (i) they have been present at HPO axis, revealed by our previous study; (ii) their pharmacological effects are related to menopause. As it has been reported that along with aging and menopause, the antioxidant enzymes is down-regulated, and the estrogen secretion through aromatase is hampered [6]. These four chemical markers possess antioxidant activities [10–13]. Besides, ferulic acid were also reported to have estrogenic properties, its treatment increases the bone mineral density in ovariectomized female rats of the Sprague-Dawley strain with slightly increasing the serum levels of estrogen [14]. In addition, it has been shown to be effective in treating hot flashes in menopausal women [15]; (iii) the amounts of these four chemical markers in EXD-S are twofold higher than those in EXD-C, thus different decoction methods could be easily revealed by different amounts of these four markers in HPLC profile (Fig. 3). In particular, palmatine is almost 30.38-fold higher in EXD-S compared with EXD-C (Fig. 3), which will be further biologically characterized in our further experiment.

Besides chemical analysis, the effects of EXD-S and EXD-C on ovarian aromatase mRNA expression and hepatic antioxidant enzymes were evaluated, which has also been proven as the targets of EXD by our group previously [5]. As anticipated, EXD stimulated ovarian aromatase (Cyp19) expression the transcriptional level at high dose (1.76 g/kg). The up-regulation of Cyp19 mRNA level in EXD-S-treated rats was significantly almost twofold higher than that of EXD-C, which may have been due to the overall increase in bioactive components in EXD-S as revealed from the HPLC profiles. It is known that the bioactive components in EXD such as mangiferin, berberine, palmatine and jatrorrhizine possess antioxidant activities [10–13]. Besides, icariin and ferulic acid were also reported to have estrogenic properties [14, 16]. The lower amounts of these bioactive compounds in EXD-C may explain the decreased bioactivity of EXD-C in vivo. The effects of EXD-S on mRNA level of hepatic antioxidants are in line with our previous findings [5]. In our previous study, EXD could significantly up-regulate CAT expression at the transcriptional level [5]. In this study, both EXD-S and EXD-C elicited around 1.5-fold of increase in the mRNA level of CAT, although no statistical significance was detected. Consistent with the results of Cyp19 expression, EXD-S showed a stronger tendency of stimulation of CAT and GPx-1 than EXD-C. The mRNA level of SOD-1 in EXD-S-treated group was significantly higher than that of the group treated with low dosages of EXD-C. These again support the better pharmacological properties of EXD-S than EXD-C.

In a TCM formula, the complexity of chemical components imposes difficulties in the identification of standard chemicals for quality control. The observation of differences in the chemical profiles of EXD-S and EXD-C in relation to their bioactivity has opened up the possibility of a novel and rapid approach to identify the standard chemicals in CM formulas. Since the pharmacological properties of a medicinal formula are conferred by the chemical components, which may change as a result of different decoction conditions. By comparing the HPLC profiles of the decoctions, the differentially extracted components would be those responsible for the observed discrepancy in bioactivity. This would facilitate the identification and selection of bioactive components as standard chemicals out of the complex herbal mixture. In a study on Radix *Scutellariae* (Huangqin) decoction, an increase in the amount of the bioactive compound baicalin was observed in the combined decoction [17]. In another study on Tangkuei Liu Huang Decoction, the amount of baicalin was higher  in separate decoctions than that of combined decoction [18]. These findings suggest that the bioactive components in a herbal extract can be affected by the decoction method as well as the herbal interaction between different herbs. The decoction methods would also affect the pharmacological properties. In some studies, the combined decoction may have better therapeutic efficacy and vice versa [19, 20]. Whether the component herbs of a Chinese medicinal formula should be decocted separately or in combination together depends on different individual formulas, but the decoction of herbal materials is often an inevitable process for the preparation of most of the CM prescriptions. The evaluation of chemical profiles as well as the pharmacological properties of different processing methods may indicate a novel approach for identifying the standard chemicals of the CM formula. In this study, the feasibility of such approach is evaluated by differential comparison of the HPLC profile of EXD-S and EXD-C in relation to the pharmacological properties. Eventually, this approach can be coupled with analytic techniques to identify the differentially extracted components obtained by different decoction methods.

In future developments, such approach may be polished by further validations with a more comprehensive pharmacological screening platform, and further evaluation of the feasibility of this approach can be conducted with other Chinese medicinal formulas. The four key chemicals, including mangiferine, ferulic acid, jatrorrhizine and palmatine, found in EXD could be further investigated in vitro and in vitro to identify their combined effects as a mixture of four in treating menopausal syndromes.

## Conclusions

In this study, the HPLC profiles of EXD-S and EXD-C have been evaluated with six known marker chemicals, which exerted actions on the HPO axis. All six chemicals were present at a higher level in EXD-S than in EXD-C. Four of them, including mangiferine, ferulic acid, jatrorrhizine and palmatine, were demonstrated to be suitable standard chemicals for quality control of EXD according to our novel and rapid approach. Both EXD-S and EXD-C displayed stimulatory effects on the expression of ovarian aromatase and hepatic SOD-1, with the effect of EXD-S being more potent. The changes of pharmacological activity in relation to the changes in chemical profiles of EXD decoction demonstrated the feasibility of a novel and rapid approach for identification of bioactive standard compounds from TCM formulas.

## Abbreviations

TCM: traditional Chinese medicine
EXD: Erxian decoction
EXD-S: mixtures of EXD individual herbs decoction
EXD-C: combined decoction of EXD
SD-rat: Sprague Dawley-rat
CAT: catalase
SOD-1: superoxide dismutase 1
GPx-1: glutathione peroxidase
Ct: threshold cycle
RSD: relative standard deviation
HPLC: high performance liquid chromatography
HPO: hypothalamus-pituitary-ovary
